# Guinea Pig Oxygen-Sensing and Carotid Body Functional Properties

**DOI:** 10.3389/fphys.2017.00285

**Published:** 2017-05-08

**Authors:** Elvira Gonzalez-Obeso, Inmaculada Docio, Elena Olea, Angel Cogolludo, Ana Obeso, Asuncion Rocher, Angela Gomez-Niño

**Affiliations:** ^1^Servicio de Anatomía Patológica, Hospital Clínico Universitario de ValladolidValladolid, Spain; ^2^Departamento de Bioquímica y Biología Molecular y Fisiología, Universidad de Valladolid, IBGM, CSICValladolid, Spain; ^3^CIBER de Enfermedades Respiratorias, ISCiiiSpain; ^4^Departamento de Enfermería, Universidad de Valladolid, IBGM, CSICValladolid, Spain; ^5^Departamento de Farmacología, Instituto de Investigación Sanitaria Gregorio Marañón, Universidad Complutense de MadridMadrid, Spain; ^6^Departamento de Biología Celular, Histología y Farmacología, Universidad de Valladolid, IBGM, CSICValladolid, Spain

**Keywords:** guinea pig, carotid body, oxygen sensing, hypoxia, ventilation

## Abstract

Mammals have developed different mechanisms to maintain oxygen supply to cells in response to hypoxia. One of those mechanisms, the carotid body (CB) chemoreceptors, is able to detect physiological hypoxia and generate homeostatic reflex responses, mainly ventilatory and cardiovascular. It has been reported that guinea pigs, originally from the Andes, have a reduced ventilatory response to hypoxia compared to other mammals, implying that CB are not completely functional, which has been related to genetically/epigenetically determined poor hypoxia-driven CB reflex. This study was performed to check the guinea pig CB response to hypoxia compared to the well-known rat hypoxic response. These experiments have explored ventilatory parameters breathing different gases mixtures, cardiovascular responses to acute hypoxia, *in vitro* CB response to hypoxia and other stimuli and isolated guinea pig chemoreceptor cells properties. Our findings show that guinea pigs are hypotensive and have lower arterial pO_2_ than rats, probably related to a low sympathetic tone and high hemoglobin affinity. Those characteristics could represent a higher tolerance to hypoxic environment than other rodents. We also find that although CB are hypo-functional not showing chronic hypoxia sensitization, a small percentage of isolated carotid body chemoreceptor cells contain tyrosine hydroxylase enzyme and voltage-dependent K^+^ currents and therefore can be depolarized. However hypoxia does not modify intracellular Ca^2+^ levels or catecholamine secretion. Guinea pigs are able to hyperventilate only in response to intense acute hypoxic stimulus, but hypercapnic response is similar to rats. Whether other brain areas are also activated by hypoxia in guinea pigs remains to be studied.

## Introduction

Mammals have developed different mechanisms to maintain oxygen delivery to cells in response to environmental or disease modifications that interfere with the gas flow. Among those mechanisms are the carotid body (CB) chemoreceptors, a specialized cell system able to detect physiological hypoxia and generate homeostatic responses. They are also partially responsible for the hyperventilation observed in respiratory or metabolic acidosis, acting as the main peripheral chemoreceptor (Gonzalez et al., 1994; Kumar and Prabhakar, 2012). CB are small paired organs located next to the carotid artery bifurcation irrigated by an extensive capillary network that separate clusters of carotid body chemoreceptor cells (type I cells) and sustentacular (type II cells). Sensory nerve fibers from the carotid sinus nerve innervate type I cells and carry chemoreceptor information to the brainstem (Finley and Katz, 1992) where reflex responses, mainly ventilatory and cardiovascular, originate to counteract the effects of hypoxia and/or hypercapnia-acidosis.

Guinea pigs, originally from the Andes, have a poor or no ventilatory response to hypoxia compared to other mammals (Blake and Banchero, 1985; Rivera et al., 1994; Curran et al., 1995; Yilmaz et al., 2005; Schwenke et al., 2007; Olea et al., 2012), implying that CB are not fully functional. This observation has been considered a genetic adaptation to high altitude because the high hemoglobin oxygen affinity (low P50) and the development of moderate erythrocytosis when exposed to chronic hypoxia (Turek et al., 1980) are characteristics retained when guinea pigs live at sea level (Rivera et al., 1994). Chronic hypoxia (CH) induces ventilatory sensitization and enhances ventilatory responses to acute hypoxia in rats (Caceres et al., 2007). However, Fernández et al. (2003) using the Dejours-type test, found that a short period of pure O_2_-breathing inhibited ventilation in guinea pigs comparable to that in the cat and higher than in the rat, suggesting that the guinea pig CB was sensitive to and silenced by pure O_2_ followed by a decrease in ventilation. Those studies also found that guinea pigs hyperventilated in response to hypercapnia with an increase in minute ventilation equal to, or higher than that observed in rats, showing that central chemoreceptors, that mediate most of the ventilatory response to hypercapnia (Alarie and Stock, 1988; Guyenet and Bayliss, 2015), as well as the brainstem integration mechanisms are well preserved.

Mechanisms involved in the detection of natural stimuli by the CB are not completely defined. It is known that several types of O_2_-sensitive K^+^ channels are inhibited by low PO_2_ causing type I cells depolarization and activation of voltage operated Ca^2+^ channels that trigger neurotransmitter release in rats (López Barneo et al., 1988; Peers, 1990; Gonzalez et al., 1994) but there are no physiological studies in guinea pig type I cells. We have undertaken this study running parallel experiments in guinea pigs and rats to check the guinea pig CB response to hypoxia compared to the well-established rat hypoxic response. Experiments have been performed in a descendent approach, from hypoxic ventilatory and cardiovascular responses to cellular characterization of guinea pig type I cells. Our findings indicate that although CB are hypo-trophic and hypo-functional, guinea pigs are able to respond by hyperventilating to intense hypoxia and to hypercapnia, not showing chronic hypoxic sensitization.

## Materials and methods

### Animals

Experiments were performed on male Hartley guinea pigs and Wistar rats (3–6 months old), fed with standard chow and water. Animals had free access to food and water and were maintained under controlled conditions of temperature, humidity and a stationary 12 h light–dark cycle. Experimental maneuvers for CH exposure (12–10% O_2_ in N_2_; inspired PO_2_~ 85 mmHg, equivalent to ~ 4,300 m; 15 days) have been described in previous studies (Caceres et al., 2007). After experiments, animals were euthanized by a cardiac overdose of sodium pentobarbital. Protocols were approved by the University of Valladolid Institutional Committee for Animal Care and Use following international laws and policies (European Union Directive for Protection of Vertebrates Used for Experimental and Other Scientific Ends (2010/63/EU).

### Plethysmography

Ventilation was measured in freely-moving animals by whole body plethysmography described by Olea et al. (2014) using methacrylate chambers (Emka Technologies, Paris, France; BUXCO Research Systems, Wilmington, NC, USA) continuously fluxed (2 l/min) with the desired gas mixture. Temperature was maintained within the thermo-neutral range (22–24°C). Body temperature was not constantly registered and it was assumed to be constant in both species during the plethysmography recordings. Tidal volume (TV; ml/Kg), respiratory frequency (BPM; breaths/min), and minute ventilation (MV; ml/min/Kg) were assessed. Animals breathed room air until acquiring a standard resting behavior. Basal ventilatory parameters and acute hypoxic and hypercapnic test response (12, 10, and 7% O_2_, remainder N_2_, and 5% CO_2_ in air were applied during 10 min followed by 15 min of recovery breathing air after each test; the analyzed recordings are the last 5 min of each tests) were measured. Pressure changes within the chamber reflecting tidal volume were assessed with a high-gain differential pressure transducer. Amplitude of pressure oscillations is proportionally related to TV; a calibration of the system by injections of 5 ml air into the chamber allowed a direct estimation of TV. Pressure signals were stored for visualization and offline analysis with Buxco software.

### Mean arterial blood pressure, blood gases, and glucose and lactate measurements

Mean arterial blood pressure (MABP) was recorded from animals anesthetized (Ketamine 100 mg/Kg and diazepam 2 mg/Kg i.p.), tracheostomized and ventilated with room air (CL Palmer) (60 cycles min–1 and a positive end-expiratory pressure of 2 cm H_2_O) or with hypoxic mixture (10% O_2_, and 90% N_2_). MABP was continuously monitored with a catheter inserted in the right common carotid artery. The catheter was connected to a pressure transducer (Statham) and signals stored (Power Lab 16SP; AD Instruments Castle Hill, Australia) for later analysis. In these animals, blood gases were obtained in normoxic (air) or hypoxic (10% O_2_) breathing conditions from a small (0.3 ml) blood sample (ABL, Radiometer Medical A/S, Denmark). Glucose and lactate content were measured from the same blood samples withdrawn when animals were breathing the different gas mixtures described above and analyzed with glucose (Ascensia Breeze 2, Bayer) and lactate meters (Lactate Pro, Arkray).

### Tyrosine hydroxylase immunostaining of the CB

Animals were perfused by gravity (1 m column) through the left ventricle with 150–200 ml of phosphate-buffered saline (PBS; 10 mM; pH = 7.4) at 37°C, followed by 250 ml of 4% (v/v) paraformaldehyde in 0.1 M phosphate buffer (PB; pH = 7.40) at 4°C. Carotid bifurcations were removed, CB cleaned and postfixed for 1 h in 4% paraformaldehyde in PB and transferred to 30% (w/v) sucrose in PB for cryoprotection. After embedding in Tissue-Tek® (Sakura Finetek, Zoeterwoude, The Netherlands) CB were frozen at –20°C. Serial sections 10 μm thick (Leitz Cryostat 1720) were collected in glass slides coated with 3-aminopropyltriethoxy-silane (Sigma, Spain). Sections were washed in PBS at room temperature and incubated in PBS containing 0.1% (v/v) Triton X-100 and 2% (v/v) nonimmunized goat serum (permeabilizing blocking solution) for 30 min. Incubation with the primary antibody (mouse anti-tyrosine hydroxylase; Sigma-Aldrich, Spain) at a dilution of 1: 1000 in permeabilizing blocking solution was carried out at 4°C overnight. After washing with PBS, sections were incubated with secondary antibody (goat anti-mouse–FITC; Sigma, Spain) and DAPI. Finally, sections were washed with PBS and distilled water and mounted in an aqueous-base mounting medium (Vectashil, Vector Laboratories). Negative controls were similarly incubated in absence of primary antibody. Sections were examined with fluorescence microscope (Axioscop 2, Zeiss) equipped with excitation and emission filters for FITC. Dissociated type I cells were similarly stained for TH as previously described for rat type I cells (Caceres et al., 2007). Images (10×; Phan-Neofluor) were captured with a CoolSnap camera and analyzed using Metamorph 6.3 software. Tyrosine hydroxylase-positive areas (TH-positive) and the entire area of the CB tissue were measured in each section to calculate the enzyme positive percentage area.

### Measurement of CB endogenous catecholamine content, synthesis rate and stimuli–evoked catecholamine release

For analysis of endogenous catecholamine (CA) content, organs (CB or superior cervical ganglion; SCG) were removed from anesthetized animals, glass to glass homogenized (0.1 N PCA and 0.1mM EDTA), centrifuged, and processed for HPLC analysis (Waters 600 controller pump, automatic injector Waters 717 plus Autosampler, and BAS LC-4C Amperometric Detector; Gemeni® C-18, 5 μm column, Phenomenex; Mobile phase: 25mM Na_2_HPO_4_, 0.65 mM 1-octane sodium sulfonate acid, 0.1mM EDTA, pH 3.46; MeOH 6%). Catecholamine identification was made against external standards and quantification was made with Peak Sample Data Chromatography System software (Buck Scientific, East Norwalk, CT).

General procedures for studying the ^3^H-CA synthesis rates have been previously described (Olea et al., 2014). Organs were incubated (37°C; 2 h) in Tyrode solution (in mM: NaCl, 140; KCl, 5; CaCl_2_, 2; MgCl_2_, 1.1; HEPES, 10; glucose, 5; pH 7.40) containing 30 μM of 3,5-^3^H-tyrosine (CA natural precursor; 6 Ci/mmol; Perkin Elmer) and 100 μM 6-methyl-tetrahydropterine and 1 mM ascorbic acid, cofactors for tyrosine hydroxylase and dopamine beta hydroxylase, respectively. Tissues were then washed in precursor-free Tyrode (4°C; 5 min) and homogenized. Identification of ^3^H-CA was carried out against internal standards at the same conditions as above and quantified by collecting the HPLC column effluents and scintillation counting.

To evaluate stimuli-evoked secretory response, isolated CB were incubated 2 h with ^3^H-tyrosine of high specific activity (40–50 Ci/mmol) and later transferred to vials containing the precursor-free Tyrode solution (in mM: NaCl, 116; KCl, 5; CaCl_2_, 2; MgCl_2_, 1.1; HEPES, 10; glucose, 5; NaCO_3_H, 24). The solution was equilibrated with gas mixtures containing 5% CO_2_ and different percentages of O_2_ (pH 7.40). When CB were incubated with high K^+^ solutions, osmolarity was maintained by removing an equimolar amount of NaCl. The hypercapnic-acidosis stimulus consisted in the same solution at pH 6.8 attained by bubbling with 20% CO_2_/20% O_2_, rest N_2_. Mitochondrial respiratory chain blockers (rotenone and Na-azide) and the mitochondrial uncoupler 2,4-dinitrophenol used as pharmacological stimuli were added to the incubating solution (drugs were obtained from Sigma, Madrid, Spain). Incubating solutions were renewed and collected every 10 min and their content in ^3^H-CA measured by scintillation counter. Experimental protocols and analytical procedures for measurement of ^3^H-CA release have been described in detail in previous publications (Gomez-Niño et al., 2009a).

### Measurement of cAMP

Levels of cAMP were measured according to previously described protocols (Cachero et al., 1996). CB were pre-incubated (15 min, 37°C) in Tyrode solution equilibrated with 95% O_2_/5% CO_2_ that was renewed with the same incubating solution containing 0.5 mM isobutylmethylxanthine (IBMX, Sigma-Aldrich, Spain). Incubating solutions were equilibrated with either 95% O_2_/5% CO_2_ (basal) or 7% O_2_/5% CO_2_/ 88% N_2_ (hypoxia). After 30 min. incubation, CB were weighed, homogenized in ice-cold 6% trichloroacetic acid and centrifuged (12,000 × g, 10 min, 4°C). Supernatants were extracted 3 times with water-saturated diethyl ether; the aqueous phase lyophilized and dried samples stored at −20°C until cAMP was assayed. An EIA commercial kit, following the instructions of the supplier (GE Healthcare Bio-Sciences AB, Uppsala, Sweden) was used. Levels of cAMP are expressed as pmole/mg tissue.

### Chemoreceptor cell culture and intracellular Ca^2+^ recording

CB were enzymatically dispersed and dissociated cells were plated on poly L-lysine-coated coverslips maintained in culture for up to 24 h as described previously (Gomez-Niño et al., 2009b). After fura-2 loading (10 μM; Pluronic F-127; Molecular Probes; 20°C, 1 h) coverslips were mounted in a perfusion chamber, placed on the stage of a Nikon Diaphot 300 inverted microscope and cells superfused with Tyrode solution containing 116 mm NaCl, 5 mm KCl, 1.1 mm MgCl_2_, 2 mm CaCl_2_, 25 mm NaCO_3_H, 10 mm glucose, 10 mm Hepes (pH 7.4 bubbling with 5% CO_2_; 20 O_2_; 75% N_2_). Dual wavelength measurements of fura-2 fluorescence were performed, using the two-way wavelength illumination system DX-1000 (Solamere Technology Group, Salt Lake City, Utah) with a 100 W Hg lamp as the light source (Optiquip, New York, NY). Light was focused and collected through a Nikon Fluor 40/1.30 objective. The wavelength for dye excitation was alternated between 340 and 380 nm, and fluorescence emission at 540 nm was collected with a SensiCam digital camera (PCO CCD imaging, Kelheim, Germany) driven by an Axon Imaging Workbench 4.0 (Axon Instruments, CA, USA). Hypoxia and 35 mM KCl were used as stimuli.

### Electrophysiological recordings

Guinea pig CB were enzymatically dissociated and under phase contrast microscope, isolated type I cells were identified by the rounded shape, brightness and size. Single cells were voltage-clamped and membrane currents were measured using the whole-cell configuration of the patch clamp technique by using an Axopatch-200B amplifier (Axon Instruments, Burlingame, CA, U.S.A) as previously reported (Frazziano et al., 2011). Currents were filtered at 3 kHz and digitalized with a Digidata 1200 analog-to-digital converter (Axon Instruments). Cells were superfused at 2 mL/min with an external Hepes solution containing (mmol/L): NaCl 141, KCl 4.7, MgCl_2_ 1.2, CaCl_2_ 1.8, glucose 10, and HEPES 10 (adjusted to pH 7.4 with NaOH). The composition (in mmol/L) of the pipette solution was: KCl 125, MgCl_2_ 4, MgATP 5, NaGTP 5, HEPES 10, EGTA 10, pH adjusted to 7.2 with KOH. Currents were evoked following the application of 50 ms depolarizing pulses from −60 mV to test potentials from −60 to +60 mV in 10 mV increments as described (Riesco-Fagundo et al., 2001). All the currents were normalized for cell capacitance and expressed in pA pF-1. Current-voltage relationships were constructed by measuring the currents at the end of the pulse. Cells were exposed to hypoxia for 10 min. Hypoxia was induced by bubbling the Hepes solution with 100% N_2_ to achieve an oxygen concentration of 3–4% (24 ± 1 Torr) in the chamber. All experiments were performed at room temperature (22–24°C).

### Data presentation and statistical analysis

Data are presented as mean ± S.E.M. Statistical analysis was performed by paired or unpaired Student's *t*-test and by repeated measures One-Way Analysis of Variance (ANOVA) with Dunnett's multiple comparison tests; *p* < 0.05 was considered statistically significant.

## Results

### Ventilatory response to acute hypoxic and hypercapnic tests

**Figure 1:**
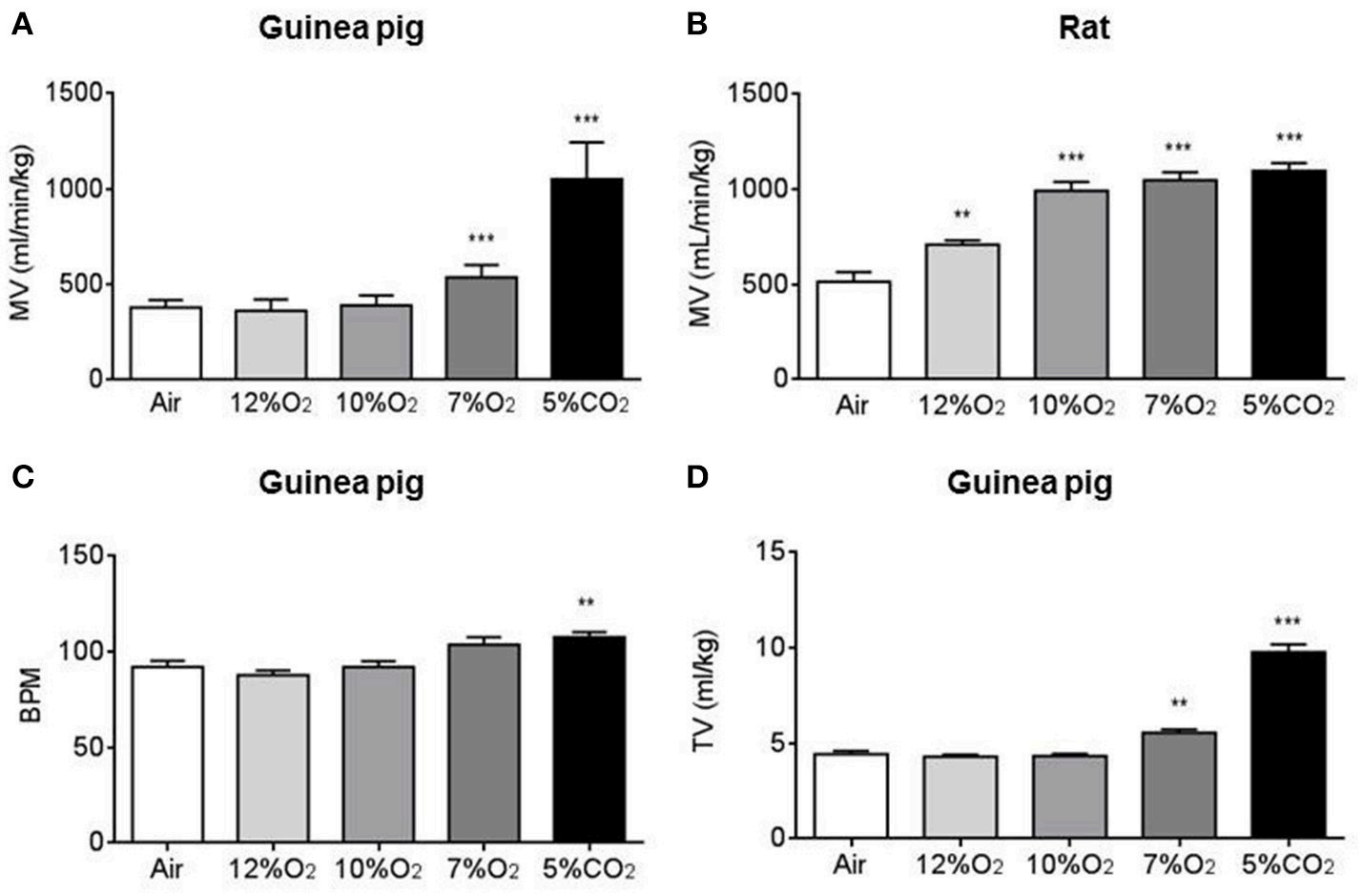
**Figure shows minute volume/Kg of weight (MV) from guinea pigs (A)** and rats **(B)** breathing air (21% O_2_), acute tests of hypoxia with different hypoxic mixtures (12, 10, and 7% O_2_) and acute test of hypercapnia (5% CO_2_). In **(C,D)** are represented the respiratory frequency (breath per min; BPM) and tidal volume (TV) from guinea pigs. Data are mean ± SEM of 16 guinea pigs and 20 rats (3–4 months old). ^**^p < 0.01 vs. air; ^***^p < 0.001 vs. air.

To assess the ventilatory response to several acute hypoxia tests (12, 10, and 7% O_2_; 10 min), continuous recording of the respiratory frequency and tidal volume (TV) were made while switching from air to the gas mixture in 16 guinea pigs and 20 rats. Figures 1 A,B shows that MV breathing room air was similar in both groups of animals. Figure also shows the effect of acute hypoxic tests on MV, increasing significantly only after intense acute hypoxic test (7% O_2_) in guinea pigs. However, MV significantly increased in rats breathing any hypoxic mixture. Figures 1C,D shows guinea pigs respiratory frequency (92 ± 3 breathing air vs. 104 ± 4 breaths/min breathing 7% O_2_) and tidal volume increasing significantly with the more intense hypoxia (4.43 ± 0.18 in air vs. 5.55 ± 0.17 ml/kg in 7% O_2_). Acute hypercapnic test (5% CO_2_ in air; 10 min) significantly increased MV in guinea pigs and rats, augmenting ventilatory parameters similarly in both species (108 ± 3 and 137 ± 4 breaths/min; 9.80 ± 0.42 and 8.01 ± 0.21 ml/kg in guinea pigs and rats, respectively).

### Mean arterial blood pressure and blood levels of gases, lactate and glucose in normoxic and acute hypoxic conditions

To assess the effects of acute hypoxia on other cardiorespiratory and metabolic parameters, arterial blood gases and mean arterial blood pressure were measured. Table 1 shows MABP from animals breathing in normoxic (air) and hypoxic (10% O_2_; 10 min) conditions. MABP in guinea pigs (50 ± 3 mmHg) were lower than in rats (115 ± 14 mmHg) in normoxia, showing that guinea pigs are hypotensive compared to rats. Breathing the hypoxic mixture induced a decline of MABP in both species (32% decrease in guinea pigs and 56% decrease in rats), more intense in rats.

**Table 1:** **Cardiorespiratory and metabolic measurements**.

**Parameters**	**Guinea pig normoxia**	**Guinea pig acute hypoxia**	**Rat normoxia**	**Rat acute hypoxia**
MABP (mm Hg)	50 ± 3	34 ± 2^***^	115 ± 14	53 ± 4^**^
pO_2_ (mm Hg)	60 ± 3	20 ± 1^***^	85 ± 3	39 ± 2^***^
pCO_2_ (mm Hg)	32 ± 1	36 ± 3	32 ± 2	30 ± 3
Glucose (mg/dL)	140 ± 11	195 ± 17^*^	147 ± 16	229 ± 20^**^
Lactate (mmol/L)	0.92 ± 0.06	1.87 ± 0.38^*^	1.66 ± 0.17	4.8 ± 0.4^***^

*Mean arterial blood pressure (MABP) and arterial blood levels of pO_2_, pCO_2_, glucose, and lactate from guinea pigs and rats in normoxia (air) and acute hypoxia (10% O_2_; 10 min) conditions. Data are mean ± SEM; n = 8*.

* *p < 0.05*;

** *p < 0.01*;

*** *p < 0.001 vs. normoxia*.

Table 1 also shows levels of different parameters obtained from small arterial blood samples taken at the end of the normoxic or hypoxic periods to monitor pO_2_, pCO_2_, glucose, and lactate. Profiles of arterial pO_2_ are comparable in both species but in all conditions are lower in guinea pigs than in rats. Arterial blood pO_2_ was 60 ± 3 mmHg in guinea pigs and 85 ± 3 mmHg in rats breathing air. The same parameter during hypoxic breathing (10% O_2_; 10 min) decreased to 20 ± 1 mm Hg and 39 ± 2 mm Hg in guinea pigs and rats showing that guinea pigs are hypoxemic compared to rats. Values of pCO_2_ were similar when animals were breathing air (32 ± 1.7 and 32 ± 2 mm Hg in guinea pigs and rats) and there were not significant differences when breathing in hypoxic atmosphere.

In the same blood samples glucose and lactate were measured. Plasma glucose levels in normoxia were very similar in both species (140 ± 11 vs. 147 ± 16 mg/dL in guinea pigs and rats, respectively). Hypoxia augmented glucose levels to 195 ± 17 mg/dl in guinea pigs and the increase was even larger in rats (229 ± 20 mg/dL). Lactate plasma variations were in concordance with those of glucose. In guinea pigs breathing the hypoxic mixture lactate rose from 0.92 ± 0.06 to 1.87 ± 0.38 mmole/l. Normoxic lactate levels in rats were higher than in guinea pigs (1.66 ± 0.17 nmole/l) and hypoxia increased to 4.8 ± 0.4 nmole/l. These data show that hypoxia is less effective increasing blood glucose and lactate levels in guinea pigs than in rats.

### Carotid body chemoreceptor cells identification

**Figure 2:**
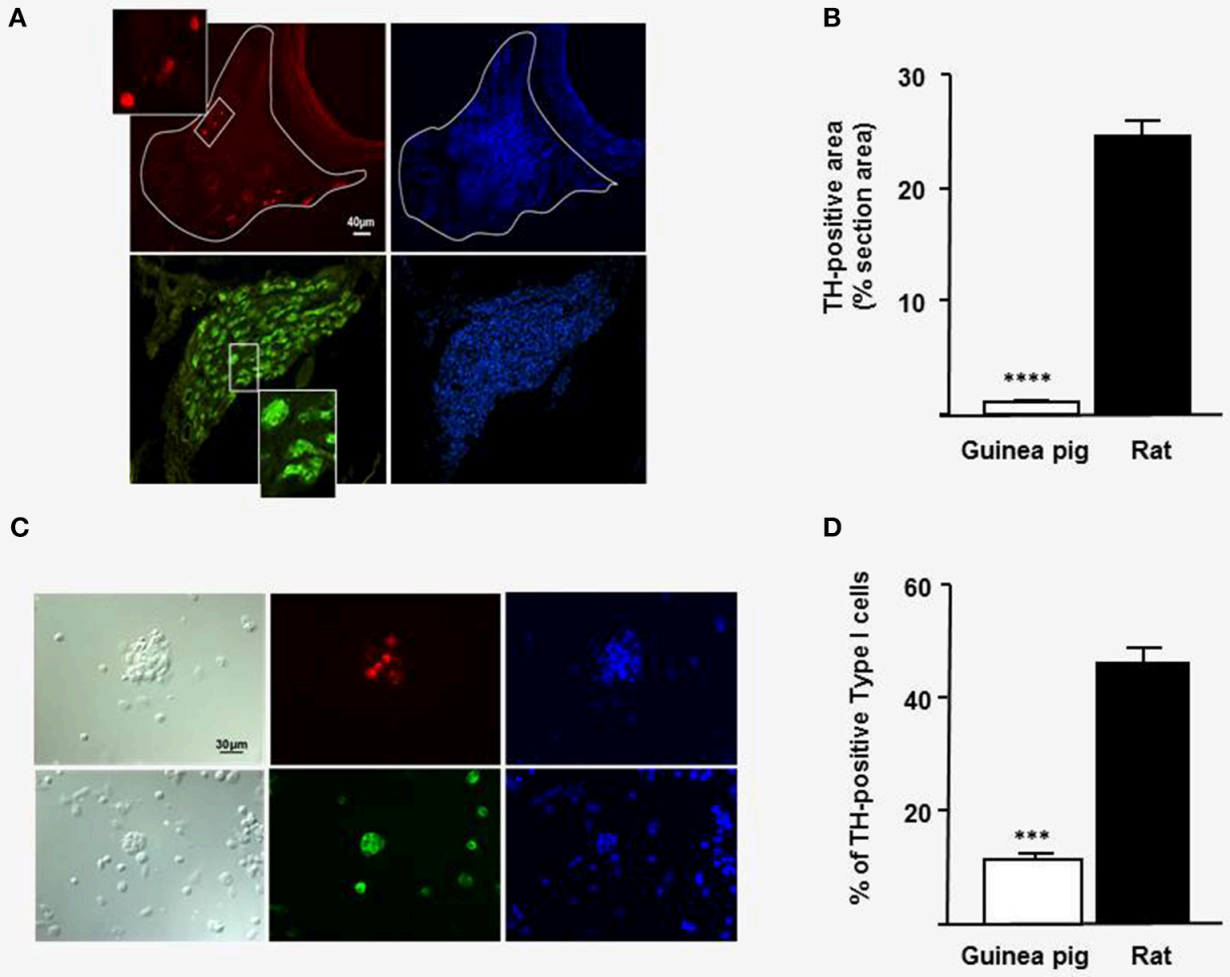
**(A)** Immunostaining for TH of central CB sections. The upper row shows a CB from guinea pig immunostaining for TH (red) and the same section immunostaining for DAPI (blue) to show cellularity. In the lower row the same sequence for rat CB (TH, green). The box shows an extension of the selected area the same scale bar for both species in **(A,C)**. **(B)** Percentage of TH-positive area obtained from CB sections from guinea pigs and rats. Data are mean ± SEM of 64 sections from 4 guinea pig CB and 150 sections from 4 rat CB (*n* = 4; ^****^p < 0.0001). **(C)** Left part shows dissociated cells from CB culture (bright field); center, immunostaining for TH and right, cell nuclei stained with DAPI. Upper row from guinea pig CB and lower row from rat CB dissociated cell culture. In **(D)** it is represented the percentage of TH-positive cells from 604 nuclei from guinea pig CB dissociated cells and 770 nuclei from rat CB dissociated cells, in both cases obtained from four guinea pigs and rats. Data are mean ± SEM (*n* = 4; ^***^p < 0.001).

Due to the poor guinea pig ventilatory response to acute hypoxia CB function was analyzed. Since CB are catecholaminergic organs immunostaining for TH was performed. Figure 2 shows CB immunostaining for TH in both species. TH immunopositive area was much smaller in guinea pig than in rat CB (Figure 2A). Quantitative TH-positive areas from 64 sections (10 μm) obtained from 4 guinea pig CB and 150 sections from 4 rat CB show the lower TH-positive area in guinea pigs, 1.07 ± 0.05% vs. 25 ± 2% in rat of total CB area sections (*n* = 4; Figure 2B). Figure 2C shows chemoreceptor cells dissociated from CB cultures and immunostained for TH. Carotid body TH-positive type I cells obtained from 4 different cultures of 4 guinea pigs (604 cells nuclei;) and from 4 different cultures of 4 rats (770 cells nuclei;) were counted. The percentage of TH-positive CB type I cells was 11 ± 2.4% and 45 ± 4.8% in guinea pig and rats, respectively (*n* = 4; Figure 2D).These data indicate that the number of chemoreceptor cells is much lower in guinea pigs than in rats.

### CA content, synthesis rate, turnover, and secretory response from guinea pig CB

The small percentage of chemoreceptor cells present in guinea pig CB drove us to investigate their functional properties. Endogenous CA content is shown in Table 2. Norepinephrine (NE) and dopamine (DA) levels from guinea pig CB in normoxia were 0.80 ± 0.10 and 1.6 ± 0.4 pmole/CB, much lower than in rat (4.57 ± 0.32 and 16.27 ± 1.47 pmole/CB of NE and DA, respectively). Since CB weight is slightly higher in guinea pigs than in rats (54 ± 2 μg in guinea pig and 48 ± 2 μg in rat), it can be estimated that guinea pig CB has approximately 1/6 of NE content and less than 1/10 of DA content compared to rat CB, according with the density of TH-positive CB cells.

**Table 2:** **Content, synthesis and turnover rate of CA from CB and superior cervical ganglion (SCG) from guinea pigs and rats in normoxia and chronic hypoxia conditions**.

		**Carotid body**				**Superior cervical ganglion**			
		**Guinea pig**		**Rat**		**Guinea pig**		**Rat**	
		**Normoxia**	**Chronic Hypoxia**	**Normoxia**	**Chronic Hypoxia**	**Normoxia**	**Chronic Hypoxia**	**Normoxia**	**Chronic Hypoxia**
CA content (pmole/CB)	NE	0.8 ± 0.1	1.15 ± 0.1^*^	4.57 ± 0.32	19 ± 6^***^	62.4 ± 4.5	94.4 ± 11.5^**^	107.3 ± 4.56	119.4 ± 2.8
	DA	1.6 ± 0.4	10 ± 0.8^***^	16.27 ± 1.47	110 ± 11^***^	48.8 ± 6.1	52.5 ± 4.5	8.3 ± 1.89	11.2 ± 2.1
^3^H-CA Synthesis (pmole/CB/2h)	^3^H-NE	0.23 ± 0.01	0.35 ± 0.02	0.18 ± 0.02	0.4 ± 0.01^***^	5.3 ± 0.5	10.9 ± 1.5^**^	2.8 ± 0.2	2.0 ± 0.3
	^3^H-DA	0.16 ± 0.01	0.89 ± 0.03^***^	2.8 ± 0.04	11.1 ± 0.5^***^	5.9 ± 0.9	11.4 ± 1.8^**^	1.7 ± 0.2	1.9 ± 0.1
Tournover rate (h)	NE	6.7 ± 1.8	6.4 ± 0.9	49.9 ± 3.9	95.8 ± 8.8^***^	11.8 ± 0.5	8.4 ± 0.5^*^	37.5 ± 1.2	59.4 ± 1.4^**^
	DA	19.4 ± 2.9	21.9 ± 2.2	11.2 ± 1.1	19.8 ± 1^**^	8.5 ± 0.4	4.6 ± 0.3^***^	5.8 ± 1.1	6.0 ± 0.7

*Data are Mean ± SEM of 8–12 data*;

** *p < 0.01*,

*** *p < 0.001 normoxia vs. chronic hypoxia*.

Chronic hypoxia increased NE content to 1.2 pmole and DA content to 10 pmole in guinea pig CB. The increase induced by chronic hypoxia in rats was approximately 4 times for NE and 7 times for DA. Table 2 also shows NE and DA synthesis rate from both species measured in normoxia and chronic hypoxia. CB from guinea pigs breathing air had a synthesis rate of ^3^H-NE and ^3^H-DA of 0.23 and 0.16 pmol/CB/2h. Values were 0.18 and 2.8 pmole/CB/2h in rats breathing air. Chronic hypoxia exposure (10% O_2_; 15 days) induced a ^3^H-NE and ^3^H-DA synthesis rate augmentation of 0.35 and 0.89 pmole/CB/2h in guinea pigs and synthesis rate increased to 0.4 and 11 pmole/CB/2h of ^3^H-NE and ^3^H-DA, respectively, in rats. In both species ^3^H-DA synthesis increased significantly in chronic hypoxic conditions while ^3^H-NE synthesis increased significantly only in rat CB. The turnover rate (content/synthesis rate) of DA and NE shows no change in guinea pigs but was double in rats CB exposed to chronic hypoxia. These data show that guinea pig CB contained 10 times less DA than rat CB and it used it faster than rat; it also contained 1/6 of rat NE and used it slightly faster than rat per time unit, probably using NE that would be contained in intraglomic sympathetic nerve endings. Since an important part of NE in the CB is contained in sympathetic nerve endings from SCG (Mir et al., 1982; Chen et al., 1997) the same CA parameters measured in CB and under the same conditions were studied in SCG (Table 2). Data showed that content and synthesis rate of NE from guinea pigs exposed to chronic hypoxia are increased and there is a 30% decrease of turnover rate, implying that sympathetic nerve endings would be pouring more NE to blood in chronic hypoxic animals. A similar decrease (50%) happened with DA turnover rate. Conversely, NE and DA content and synthesis are not significantly modified in rat SCG after chronic hypoxic treatment.

**Figure 3:**
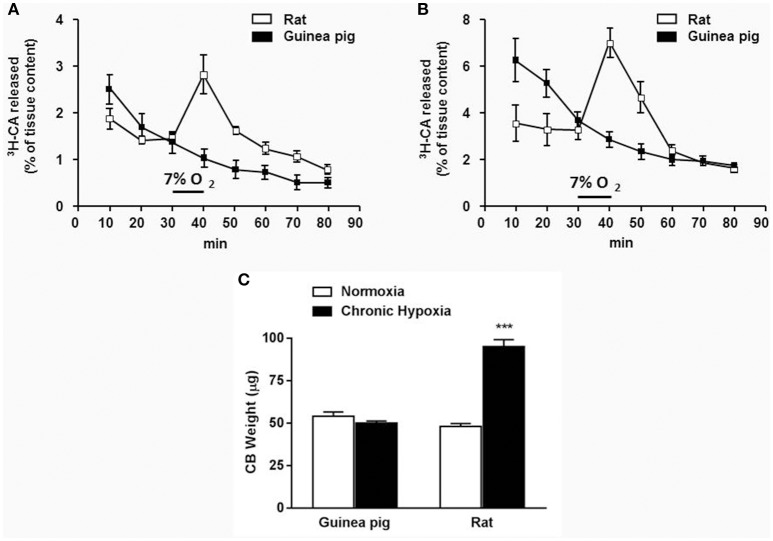
**Guinea pig and rat time course of ^3^H-CA secretion elicited by 7% O_2_ from normoxic (A)** and chronic hypoxic **(B)** CB. Data are expressed as mean ± SEM; *n* = 8–12. **(C)** Guinea pig and rat CB weight from normoxic and chronic hypoxic animals (exposed to 10–12% O_2_; 15 days). Data are mean ± SEM of 60 normoxic and 34 chronic hypoxic guinea pigs CB and of 60 normoxic and 48 chronic hypoxic rats CB. ^***^p < 0.001 chronic hypoxia vs normoxia.

It is known that hypoxia and other natural and pharmacological stimuli activate CB inducing the release of CA from mouse, rat, rabbit, and cat CB (Gonzalez et al., 1994). This activity index was used to measure CA secretion from guinea pig CB in response to different stimuli. Figure 3 shows that hypoxia (7% O_2_) did not modify ^3^H-CA evoked release from neither normoxia nor chronic hypoxia isolated guinea pig CB. Conversely, the same stimulus evoked a ^3^H-CA release of 3.2% ± 0.3 of tissue content from normoxic rat CB that was even larger in chronic hypoxia rat CB (7.1% ± 0.6). As shown in Figure 3C, CH exposure, a condition that sensitize CB and induce CB hypertrophy in other species did not modify guinea pig CB size, agreeing with the lack of CB sensitization to hypoxia.

The same lack of guinea pig CB ^3^H-CA evoked release response was found when other natural (2% O_2_, hypercapnia/acidosis; Table 3) or the classical pharmacological stimuli (mitochondrial respiratory chain blockers rotenone 1 μM and Na-azide 5 mM or the uncoupler DNP 0.5 mM) were used (data not shown). Only the non-specific depolarizing high extracellular K^+^ stimulus was able to induce a ^3^H-CA release response comparable in both species. However, when all those stimuli were applied to normoxic or chronic hypoxic rat CB a significant increase of CA release response was observed (Gomez-Niño et al., 2009a).

**Table 3:** **Normoxia (air) and chronic hypoxia (10–12% O_2_; 15 days) ^3^H-CA release response from guinea pig and rat CB induced by different natural stimuli and high extracellular K^+^**.

**CB stimulus**	**^3^H-CA evoked release (% of tissue content)**			
	**Guinea pig**		**Rat**	
	**Normoxia**	**Chronic hypoxia**	**Normoxia**	**Chronic hypoxia**
Mild hypoxia (7% O_2_)	0 (*n* = 12)	0 (*n* = 8)	3.2 ± 0.3 (*n* = 18)	7.1 ± 0.6^***^ (*n* = 8)
Severe hypoxia (2% O_2_)	0 (*n* = 12)	0.2 ± 0.2 (*n* = 12)	10.1 ± 1.3 (*n* = 12)	17.2 ± 1.6^***^ (*n* = 8)
Acidosis/hypercapnia (pH = 6.8)	0 (*n* = 12)	0 (*n* = 8)	0.3 ± 0.1 (*n* = 12)	0.35 ± 0.1 (*n* = 6)
35 mM K^+^	3.3 ± 0.2 (*n* = 12)	7.2 ± 0.9^***^ (*n* = 12)	4.2 ± 0.6 (*n* = 6)	14.7 ± 1.3^***^ (*n* = 8)

*** *p < 0.001 chronic hypoxia vs. normoxia, implying sensitization of the CA release response*.

### Effect of hypoxia and high external K^+^ on intracellular Ca^2+^ levels from type I cells

**Figure 4:**
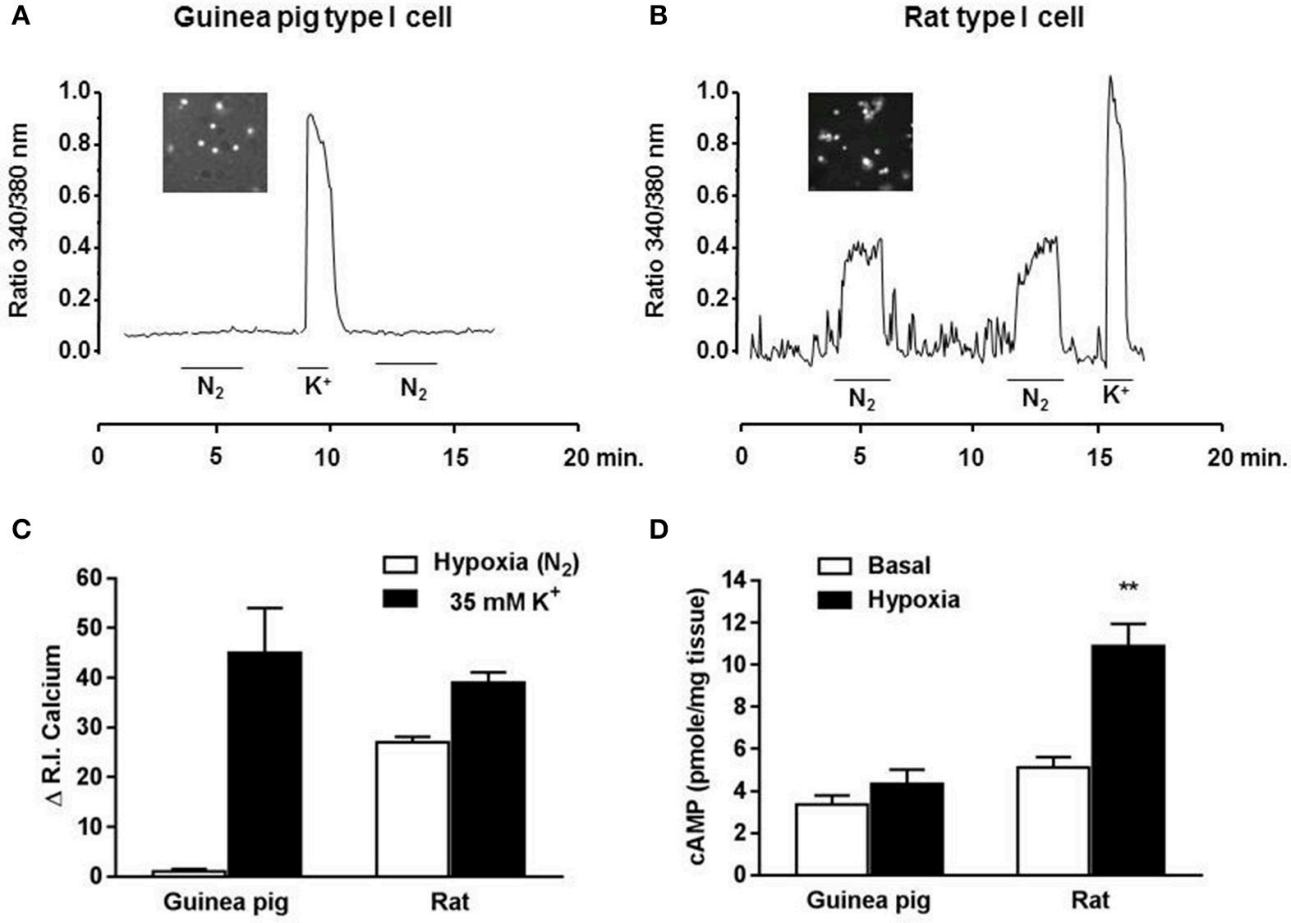
**Intracellular Ca^2+^ response to hypoxia and high external K^+^ in isolated chemoreceptor cells from guinea pig and rat CB**. **(A)** Sample recording obtained in a guinea pig type I cells from the microscope field (insert) showing the lack of response to the hypoxic stimuli and the increase elicited by perfusion with 35 mM K^+^ solution. **(B)** Shows the oscillatory behavior of the intracellular Ca^2+^ levels in normoxia and the moderate sustained increase elicited by a hypoxic solution and by perfusion with 35 mM K^+^ solution from a rat type I cells. In every cell the fluorescence signal was integrated as a function of time (running integral; RI), showed in **(C)**. Mean ± SEM of the RI in 16 type I cells from 3 guinea pigs and 29 type I cells from 2 rats. **(D)** Levels of cAMP from guinea pig and rat CB in normoxic and acute hypoxic conditions. Data expressed as mean ± SEM; *n* = 6; ^**^p < 0.01 vs. normoxia.

Figure 4 shows the changes in intracellular Ca^2+^ levels from type I cells obtained from 3 guinea pig and 2 rat CB in response to hypoxia and high extracellular K^+^. Hypoxia did not change the intracellular Ca^2+^ levels in guinea pig isolated type I cells, while the same hypoxic exposure led to reproducible increases in Ca^2+^ levels from isolated rat type I cells. However, TH-positive cells from both, guinea pig and rat CB, when challenged with a pulse of 35 mM K^+^, responded with a brisk increase in intracellular Ca^2+^ which reached comparable peaks, followed a similar time course and exhibited an overall similar integrated amplitude. Figure 4D shows acute hypoxic effects (7% O_2_) on levels of cAMP obtained from guinea pig and rat CB. Levels of cAMP were significantly increased after hypoxia exposure in rat CB but not in guinea pig CB, implying a lack of this second messenger pathway activation by hypoxia.

### Electrophysiological recordings from guinea pig and rat isolated type I cells

**Figure 5:**
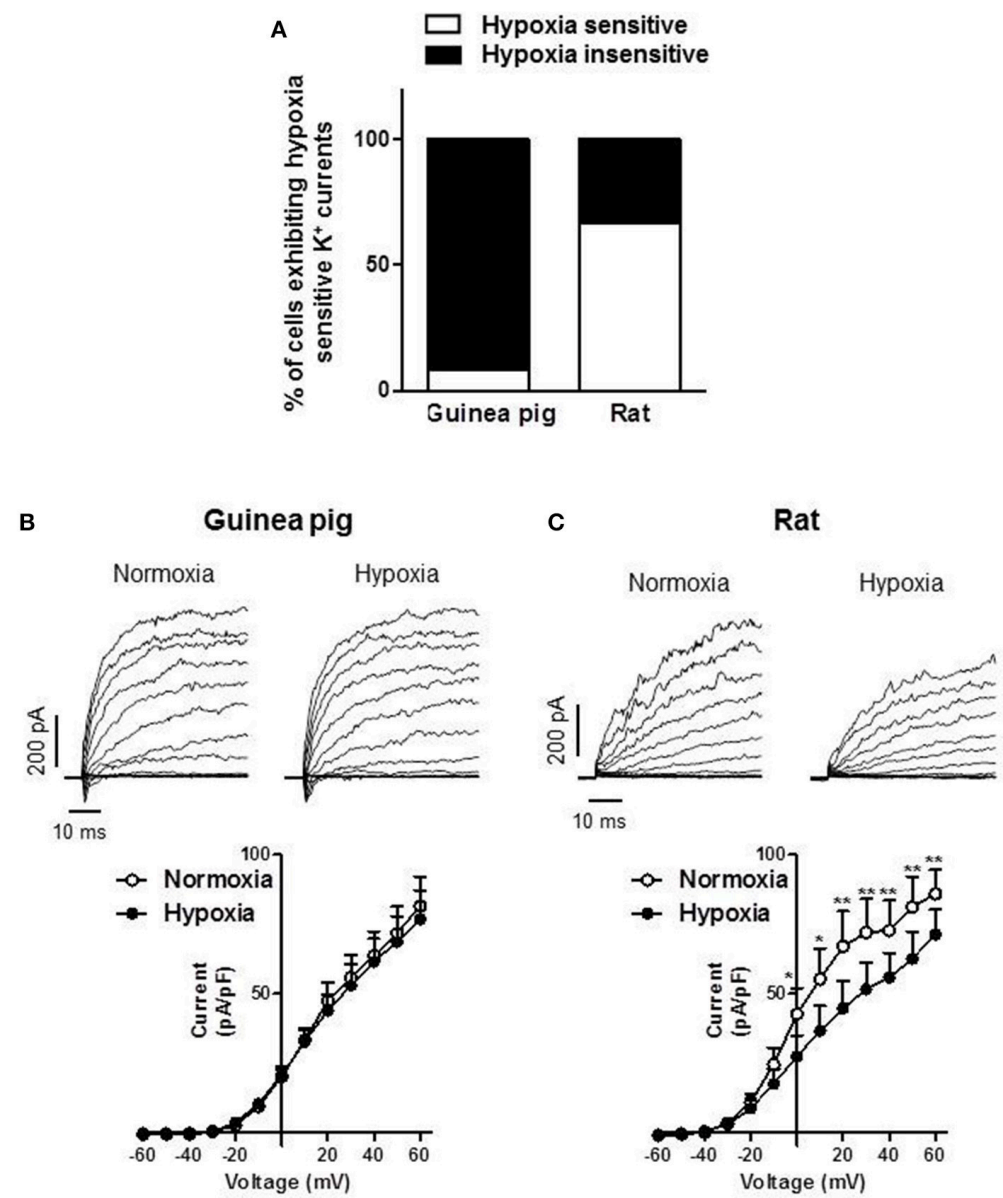
**Sensitivity of K^+^ currents to hypoxia in guinea pig vs. rat type I cells. (A)** Percentage of cells with hypoxic sensitive or insensitive K^+^ currents isolated from guinea pig and rat CB. **(B)** Representative potassium current traces for 50 ms depolarization pulses from −60 to +60 mV in 10 mV increments from a holding potential of −60 mV in isolated type I cells from 3 guinea pigs. **(B)** and 2 rats. **(C)** Superfused with normoxic (air-equilibrated) and hypoxic (N_2_-equilibrated) conditions. Current-voltage relationships of K^+^ currents (I-V curves) are represented below. Data are expressed as mean ± SEM; ^*^, ^**^p < 0.05 and 0.01 vs. normoxia.

The inhibition of K^+^ channels is considered an initial step in the response of type I cells to a decrease in PO_2_ (López Barneo et al., 1988; Peers, 1990; López-López et al., 1997). We examined the presence of this first element of the transduction cascade on guinea pig and rat type I cells. We found that the majority of the guinea pig CB dissociated cells were insensitive to hypoxia (11 out of 12 cells from 3 different animals (Figure 5A). Conversely, hypoxia inhibited K^+^ currents in most of rat type I cells (6 out of 9 cells from 2 different animals, Figure 5A). Figures 5B,C show representative experiments of hypoxic insensitive guinea pig CB cells and hypoxic sensitive rat CB cells, respectively. The current voltage relationships of these representative experiments are also shown. The inhibitory effect of hypoxia in rat type I cells reached statistical significance at potentials more positive than −10 mV. Only one of the guinea pig chemoreceptor cells responded to hypoxia with an inhibition of the K^+^ currents at +60 mV of about 25%.

## Discussion

The main goal of this study was to understand how guinea pigs defend against hypoxia. Plethysmography data show that guinea pig and rat ventilatory parameters breathing air are similar but guinea pigs hyperventilate only in response to intense hypoxic test (7% O_2_) not showing ventilatory changes when breathing 12 or 10% O_2_ atmosphere (mild hypoxia). All those hypoxic conditions induced a significant increase of ventilation in rats. A potential weakness of these data could be the lack of body temperature fluctuation correction that perhaps could underestimate TV, and as a result, modify the O_2_ threshold level for the hypoxic response in both species and it could be possible that guinea pigs (and rats) increase their MV at milder levels of hypoxia. However, other results from guinea pig *in vitro* CB showing the same lack of hypoxic response overrode this possibility. Severe hypoxic stimulus (8% O_2_) used by Schwenke et al. (2007) did not detect any increase of guinea pig carotid sinus nerve activity and denervation did not affect ventilation nor cardiovascular variables. It could be that CB hypoxic detection/transduction or other elements of the chemoreceptor reflex responsible for the ventilatory response to hypoxia are not completely functional in guinea pigs and/or the activation threshold is higher. Tidal volume and minute ventilation in mild hypoxic conditions in this study were similar to those described by other authors (Arold et al., 2002; Yilmaz et al., 2005; Olea et al., 2012). However, acute hypercapnic test increased MV similarly in both species and inspiratory and expiratory normoxic flows were also comparable. The observed hypercapnic response, generated partially by CB and mainly by central chemoreceptors (Gonzalez et al., 1994; Guyenet and Bayliss, 2015), suggests that guinea pig CB and central projections are functional, as was also demonstrated by Schwenke et al. (2007) after denervating guinea pig CB and preserving 72% of acute hypercapnic (8% CO_2_) response. The similar response to hypercapnia in both species would imply that guinea pig afferent integration of CB and central chemoreceptors in the respiratory center are normal or alternatively, that guinea pig central chemoreceptors supplied hypercapnic CB contribution.

Macroscopic observation of guinea pig and rat CB shows that size is similar although CB weight related to body weight is smaller in guinea pigs. (3 months old guinea pigs weight = 618 ± 11 g; *n* = 16 and rats weight = 316 ± 8; *n* = 24). When rats were exposed to CH during 15 or more days, CB weight increased about 2 times but guinea pig CB was not modified or it was slightly smaller under the same conditions. This response to CH increasing CB size is also observed in other mammals from mouse to human (Heath and Smith, 1992; Wang and Bisgard, 2002). Patients that died from hypoxemic diseases also have hypertrophic CB (Heath and Smith, 1992) mainly due to an increase of CB vessels (Laidler and Kay, 1975; Del Rio et al., 2011) apparently caused by an augmented VEGF production in chemoreceptor cells (Wang and Bisgard, 2002; Tipoe and Fung, 2003). It has also been related to an increased endothelin-1 production and type I cells proliferation (Platero-Luengo et al., 2014). It could be that CH do not induce an increase of VEGF in guinea pig CB. However, guinea pig hematocrit significantly increased after CH exposure (5% increase; Olea et al., 2012) but also less than in rats (15% increase), indicating that HIF-1α, the controlling transcription factor for EPO and VEGF expression during hypoxia is active (Semenza, 2006). Immunocytochemical and immunohistochemical data show a limited number of TH-positive cells in CB sections or isolated guinea pig type I cells compared to rat. Quantitative TH-positive areas measured in CB sections is 20 times lower in guinea pig than in rat (1 and 25%, respectively, from total area) and TH-positive cells are also 4 times lower in guinea pigs than in rats (11 and 45%, respectively, from total DAPI positive nuclei). Therefore, guinea pig CB is hypotrophic and with lower number of TH-positive type I cells compared to rat CB. Kummer et al. (1990) described heterogeneity of guinea pig chemoreceptor cells with respect to TH immunoreactivity suggesting the existence of distinct type I cells in this species.

CA metabolism in chemoreceptor cells has been used as an index of CB activity, because synthesis and release of CA increase proportionally to the intensity of the stimuli (Gonzalez et al., 1994). In this study guinea pig and rat CB are dopaminergic organs, but CA (DA + NE) content and synthesis rate in guinea pig CB is lower than in rat. It has been described that exposure to CH augment TH expression, the rate limiting enzyme of CA synthesis (Wang and Bisgard, 2002), which agree with the increase of the CA synthesis rate in both species, showing that guinea pig CB was stimulated. In SCG, NE levels in guinea pigs were half of that in rats, suggesting a lower noradrenergic tone and also, unusually high levels of DA, a negative modulator of sympathetic transmission. NE is the sympathetic nervous system neurotransmitter responsible for the resting tonic maintenance of cardiovascular function. Guinea pigs are markedly hypotensive compared to rats and this could be related to the lower noradrenergic tone. The low arterial pressure and low arterial pO_2_ in guinea pigs are not related to hypoventilation since respiratory frequency was not significantly modified when animals were breathing air or 12 and 10% O_2_, but it increased when breathing 7% O_2_ and 5% CO_2_. Low arterial pressure has also been related to higher capillarity in peripheral tissues and it has been observed that guinea pigs have a higher capillarity density in pulmonary circulation than rats (Schraufnagel and Schmid, 1988; Sekhon et al., 1995). Guinea pigs, even if adapted, when living at sea level maintain the genetic traits of a highlander (Winslow, 2007; Pairet and Jaenicke, 2010).

Recently, new roles for CB have been proposed, showing that the tonic chemoreceptor drive may have a function in the development of sympathetic over-activity in hypertension (Siński et al., 2012; Del Rio et al., 2015). It could also be that guinea pigs' low arterial pressure is related to a lower sympathetic drive as a result of a reduced CB function in this species. Several natural or pharmacological stimuli were unable to activate CA secretory response from *in vitro* guinea pig CB but all of them activated the secretory response from rat CB. Only high extracellular K^+^ a non-specific stimulus induced a comparable CA secretory response in both species, impling that chemoreceptor cells can be excitated and that the exocytotic machinery is functional in guinea pig CB. CH induced a more intense response to high extracellular K^+^, sensitizing CB to this depolarizing stimulus. This could be due to a higher CA content or to sensitization of the exocytotic mechanisms, so that to similar intracellular Ca^2+^ concentration exocytosis is more efficient (Ghijsen and Leenders, 2005). Alternatively, a remodeling of L-type Ca^2+^ channels implied in this secretory response would explain the enhanced response (Rocher et al., 2005). In hypoxic conditions there was not any modification in the transients of intracellular Ca^2+^ from isolated guinea pig type I cells but high extracellular K^+^ increased it noticeably. Conversely, the same hypoxic stimulus produced a change of fluorescence approximately half of that of high K^+^ in rat chemoreceptor cells. These observations would indicate that guinea pig chemoreceptor cells express voltage-dependent Ca^2+^ channels activated when cells are depolarized but hypoxia is not able to activate them (Rocher et al., 2005). Similarly, increased cAMP levels in response to acute hypoxia has been involved in the O_2_-sensing/transduction mechanism in type I cells (Rocher et al., 2009). cAMP activate EPAC acting as an amplifier mechanisms in rat type I cells exocytosis as it has been already described (Rocher et al., 2009). Lack of increased cAMP levels induced by acute hypoxia in guinea pig CB could explain the lack of hypoxic induced CA secretory response. The lack of an increase of Ca^2+^ levels induced by hypoxia makes it unlikely that other neurotransmitters could be implicated in the guinea pig CB secretory response.

It is accepted that several types of K^+^ channels sensitive to hypoxia are the first effector component of the transduction cascade in chemoreceptor cells of all studied species. We show for the first time that guinea pig type I cells have a K^+^ voltage-dependent current density very similar to rats. Interestingly, while the K^+^ currents were inhibited by hypoxia in most of the rat type I cells tested (66%), this effect was only observed in one of the type I cells guinea pig tested (9%). These data are in agreement with the lack of hypoxia induced Ca^2+^ and cAMP levels increase, CA secretory response and the low TH-positive type I cells from guinea pigs. Altogether, these data imply that the main mechanisms to respond to acute hypoxia in the CB are not fully functional in guinea pigs.

There are two adaptive mechanisms of almost instant appearance in acute hypoxia, hyperventilation mediated by the CB and pulmonary hypoxic vasoconstriction. Moderately elevated pulmonary artery pressure enhances systemic oxygen delivery by increasing blood flow into lung areas with relatively lower blood flow, improving the total alveolar capillary surface area for gas exchange. Pulmonary hypoxic vasoconstriction is limited in guinea pigs compared to rats (Swenson, 2013). Maintained low PO_2_ starts a third mechanism, the increase of erythropoiesis. The erythropoiesis induced by chronic hypoxia is also scant in guinea pigs (Olea et al., 2012) compared to rats. Rivera et al. (1994) studying the ventilatory response to severe acute hypoxia in guinea pigs and rats with high hemoglobin-oxygen affinity induced by cyanate found that baseline ventilation, hemoglobin concentration and P50 were significantly lower in guinea-pigs than in rats, concluding that guinea pigs probably use tissue and biochemical adaptive mechanisms to successfully tolerate ambient hypoxia. Hemoglobin is involved in the regulation of O_2_ transport in specialized species native to high altitudes and guinea pigs seem to have a lower P50 than their sea level counterparts, an adaptation that presumably promotes O_2_ uptake from a hypoxic environment (Winslow, 2007). Another characteristic of high altitude inhabitants, the relatively low arterial blood pressure is also shown by guinea-pigs. It could be that adaptation to acute and chronic hypoxia in high altitude native animals is based in changes of O_2_ hemoglobin affinity (Frappell et al., 2007; Winslow, 2007; Pairet and Jaenicke, 2010). It has been published that guinea pig hemoglobin has a higher O_2_ affinity than mammals of similar size living at sea level (Yilmaz et al., 2005; Pairet and Jaenicke, 2010). Several studies have identified regions of the caudal hypothalamus and rostral ventrolateral medulla in a number of species that are directly excited by hypoxia and when activated, increase sympathetic discharges and cause increase of blood pressure and heart rate (Guyenet, 2000; Neubauer and Sunderram, 2004; Mandel and Schreihofer, 2009; King et al., 2015). The possible presence of oxygen-chemosensitive catecholaminergic neurons distributed throughout the brain stem may form an oxygen-chemosensitive network that could also contribute to maintain guinea pigs arterial oxygen levels during hypoxic stimuli, in spite of the hypofunctional guinea pig CB.

In summary, guinea pigs are able to hyperventilate in response to intense hypoxic stimulus in spite of the hypotrophy, small percentage of TH-positive cells and lack of response of carotid body chemoreceptor cells to hypoxic challenge. Nevertheless, guinea pigs show a higher tolerance to hypoxic environment than other rodents. Whether additional chemoreceptor brain areas are also activated by hypoxia remains to be studied.

## Author contributions

Conception and design of the work, AO, AR, and AG; Acquisition and interpretation of data, EG, ID, EO, AC, AO, AR, AG; Statistical analysis of the data, EO, ID, AC, AR, and AG; Drafting of manuscript and revising critically, EG, AO, AC, AR, and AG; Final approval of the version to be published, EG, ID, EO, AC, AO, AR, and AG.

## Funding

This study was supported by grants BFU2015-70616R, SAF 2014-55399 and SAF 2016-77222-R (MINECO-FEDER), and CIBER CB06/06/0050 (ISCiii).

### Conflict of interest statement

The authors declare that the research was conducted in the absence of any commercial or financial relationships that could be construed as a potential conflict of interest.
